# The Development of a Critical Care Resident Research Curriculum: A Needs Assessment

**DOI:** 10.1155/2016/9795739

**Published:** 2016-08-16

**Authors:** Sangeeta Jain, Kusum Menon, Dominique Piquette, Ronald Gottesman, James Hutchison, Elaine Gilfoyle, Canadian Critical Care Trials Group

**Affiliations:** ^1^Department of Pediatrics, 2888 Shaganappi Trail NW, Calgary, AB, Canada T3B 6A8; ^2^Children's Hospital of Eastern Ontario, 401 Smyth Road, Room 3446, Ottawa, ON, Canada K1H 8L1; ^3^Sunnybrook Health Sciences Centre, 2075 Bayview Avenue, Room D108, Toronto, ON, Canada M4N 3M5; ^4^Division of Critical Care, Department of Pediatrics, Montreal Children's Hospital, 1001 Decarie Boulevard, Room B06.3834.2, Montreal, QC, Canada H4A 3J1; ^5^Neuroscience and Mental Health Research Program, Hospital for Sick Children Research Institute, 555 University Avenue, Toronto, ON, Canada M5G 1X8; ^6^Section of Critical Care, Department of Pediatrics, Alberta Children's Hospital, 2888 Shaganappi Trail NW, Calgary, AB, Canada T3B 6A8; ^7^Centre de Recherche du CHUM, Tour Viger, 900 rue Saint-Denis, Room R04-470, Montreal, QC, Canada H2X 0A9

## Abstract

*Background*. Conducting research is expected from many clinicians' professional profile, yet many do not have advanced research degrees. Research training during residency is variable amongst institutions and research education needs of trainees are not well understood.* Objective*. To understand needs of critical care trainees regarding research education.* Methods*. Canadian critical care trainees, new critical care faculty, program directors, and research coordinators were surveyed regarding research training, research expectations, and support within their programs.* Results*. Critical care trainees and junior faculty members highlighted many gaps in research knowledge and skills. In contrast, critical care program directors felt that trainees were prepared to undertake research careers. Major differences in opinion amongst program directors and other respondent groups exist regarding preparation for designing a study, navigating research ethics board applications, and managing a research budget.* Conclusion*. We demonstrated that Canadian critical care trainees and junior faculty reported gaps in knowledge in all areas of research. There was disagreement amongst trainees, junior faculty, research coordinators, and program directors regarding learning needs. Results from this needs assessment will be used to help redesign the education program of the Canadian Critical Care Trials Group to complement local research training offered for critical care trainees.

## 1. Introduction

Research is a mandatory activity for all Royal College of Physician and Surgeons of Canada (RCPSC) training programs, including the field of critical care. Trainees in critical care “are expected to participate in a basic or clinical research project” and must “demonstrate a basic understanding of biostatistics, study design, protocol writing, and manuscript preparation…under the direction of a scientist or Critical Care Medicine specialist” [1, 2]. This has become an integral part of training programs since we know that a better understanding of reported studies is linked to improved patient care and improvement in the academic mind of the physician [3]. In addition and on a more practical level, having published research is an advantage when applying for further training or employment [4]. Various barriers to successful research amongst trainees have been described in the literature, including lack of interest, lack of time, and poor understanding of research methods [3, 5].

In Canada, some individual residency programs organize a departmental-specific research course or have trainees who participate in university-wide research courses. These experiences are variable due to lack of resources of each individual program or courses not specific to critical care. Organizations that are not directly affiliated with official training programs may also be tasked to provide research training. The Canadian Critical Care Trials Group (CCCTG) is an internationally recognized group of critical care clinicians and researchers who collaborate to conduct high-quality critical care-based research. The CCCTG currently offers a “Resident Research Day,” where CCCTG members teach about various aspects of research (such as ethics in critical care research) followed by critical care residents and other critical care research trainees presenting their research projects for feedback from their peers and members of the CCCTG and Canadian Critical Care Translational Biology Group. This research day was designed for critical care trainees based on data from a focus group conducted with CCCTG faculty and trainee members (unpublished data). However, it has been several years since the implementation of this training curriculum; it is unknown whether there has been a change in local resources available to trainees or whether the needs of trainees have evolved. One aspect of the mission of the CCCTG is to “mentor and support aspiring investigators and future leaders in critical care research” (http://www.ccctg.ca/About-Us/Strategic-Directions.aspx).

When developing a new curriculum (or revising an existing one), performing a needs assessment is the necessary first step [6]. Without a needs assessment, any curriculum developed risks devoting unnecessary resources to areas already mastered by the learner and/or paying insufficient attention to areas of particular weakness.

Through a needs assessment, we aim to identify and describe what the learning needs are for research education in critical care in Canada. We plan to target both perceived needs (from the learners themselves) and unperceived needs (from program directors, new faculty researchers, and research coordinators) regarding trainees' needs. Specifically, we aim to gain insight into (1) perceived and unperceived knowledge gaps about the research process, (2) barriers to successful research during residency training, and (3) tools and skills that would be useful for trainees before they take on faculty positions with research requirements. The ultimate aim of this project is to obtain information useful for the development of a research curriculum targeting trainees currently involved in research, with the objective to encourage participation in research as part of their future careers.

We hypothesize that there will be differences between the perceived and unperceived needs related to critical care research education, which will be highlighted when comparing responses amongst trainees, program directors, new faculty, and research coordinators.

## 2. Methods

We surveyed various stakeholders including adult and pediatric critical care residents, new academic critical care faculty, critical care research coordinators, and critical care residency program directors during the July 2012 to June 2013 training year.

A voluntary, confidential, self-administered online survey in English (via Survey Monkey®) was sent to eligible participants. Critical care residents were recruited by gathering their contact information from their program directors. We also contacted the Critical Care Department Heads across the country to identify new academic faculty (within 5 years of their appointment). Research coordinators were contacted via the Canadian Critical Care Research Coordinators Group. The research team contacted potential subjects by sending out an electronic letter explaining our research goals with a link to the survey attached. In order to maximize response rate, we emailed participants ahead of the survey and also sent two follow-up reminders after the initial survey was sent out, for a total study period of six months.

The questionnaire for trainees (see Appendix A in Supplementary Material available online at http://dx.doi.org/10.1155/2016/9795739 for the full document) consisted of 4 domains: demographic information, current research activities, previous research training (including the CCCTG research day), and a self-assessment of knowledge with various aspects of research (e.g., developing a research question). A similar survey was sent out to university-affiliated critical care faculty who are within 5 years of completing their critical care fellowship. Questionnaires were also distributed to program directors and research coordinators.

Data from Survey Monkey were downloaded to and analyzed in Microsoft Excel®. Quantitative data were analyzed with descriptive statistics (mean and median). For descriptive analyses, we used actual number of respondents for the denominator. We collapsed categories where appropriate to summarize responses in a meaningful manner. Comparisons were made between different respondents; however statistical comparisons were not pursued due to low numbers. Written comments were summarized and grouped into themes.

Ethics approval was obtained from the Conjoint Health Research Ethics Board of the University of Calgary.

## 3. Results

### 3.1. Demographics

Surveys were sent to a total of 235 potential participants with 86 completed surveys returned, yielding an overall response rate of 37%.  Table 1 describes the summary of survey responses within each category of respondent.  Table 2 describes the demographics of the critical care trainees who responded to the survey.

### 3.2. Trainee Responses

Of the trainees who responded, 60.7% identified an interest in doing research as a part of their future careers in critical care. At the time of the survey, 86% of trainees were currently involved in a research project. Of the 28 respondents, the majority (75%) had projects representing clinical research, whereas a much smaller percentage was pursuing medical education-based projects or translational medicine projects (7% and 4%, resp.).

**Figure 1:**
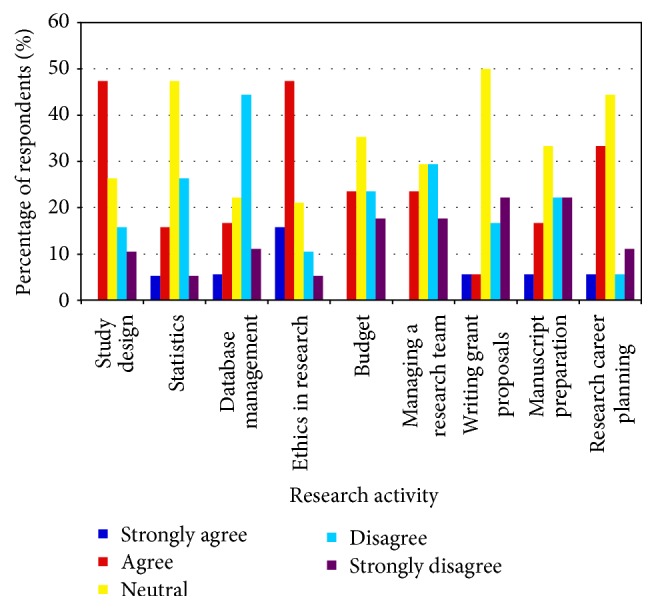
Response of critical care trainees to the question: “Formal research training during my fellowship in Critical Care has provided me with the skills to be proficient in the following areas of research.”

**Figure 2:**
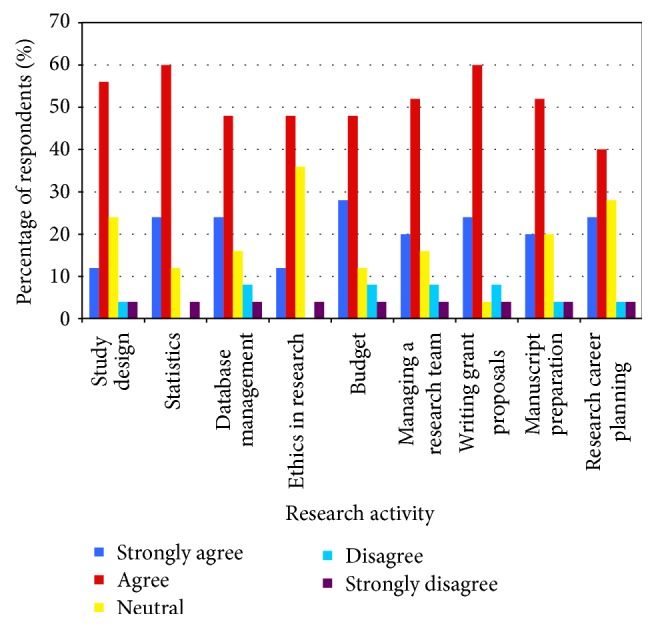
Response of critical care trainees to the survey question: “I feel that, in order to assume my future professional responsibilities, I would benefit from more training in the following areas of research.”

Trainees noted that formal research training during their critical care fellowships provided them with a good overview of study design and ethics in research but did not give them the skills to be proficient in the specific areas of database management and managing a research team (Figure 1). Overall, trainees felt that, in order to assume future professional and research responsibilities, they would benefit from more training in most areas of research, but especially in statistics and writing grant proposals (Figure 2). Trainees felt most comfortable with their ability to navigate ethics in research.

### 3.3. Faculty Member Responses

**Figure 3:**
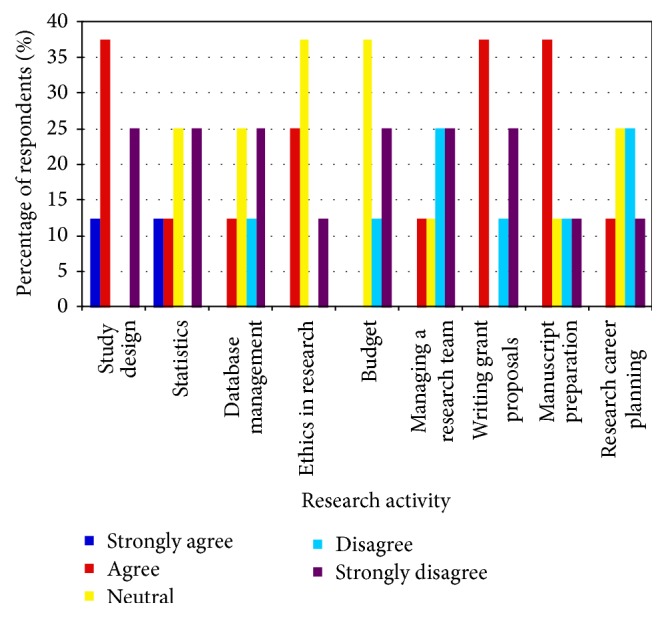
Junior faculty members' response to the question: “Formal research training during my fellowship in Critical Care provided me with the skills to be proficient in the following areas of research.”

Faculty members who responded to the survey had all been trained in Canadian critical care programs. Of faculty members surveyed, 36% had advanced degrees in areas such as epidemiology, public health, and health administration. Eighteen percent of respondents spent a majority of their nonclinical time on research and had protected time to do so. Forty percent of faculty participated in formal research education outside of the CCCTG Research Day. In these research education programs, faculty identified areas of research not generally touched upon: managing a database management, developing budgets, and writing grant proposals. After formal training, faculty members still did not feel proficient in database management, creating a research budget or managing a research team (Figure 3).

### 3.4. Program Director Responses

**Figure 4:**
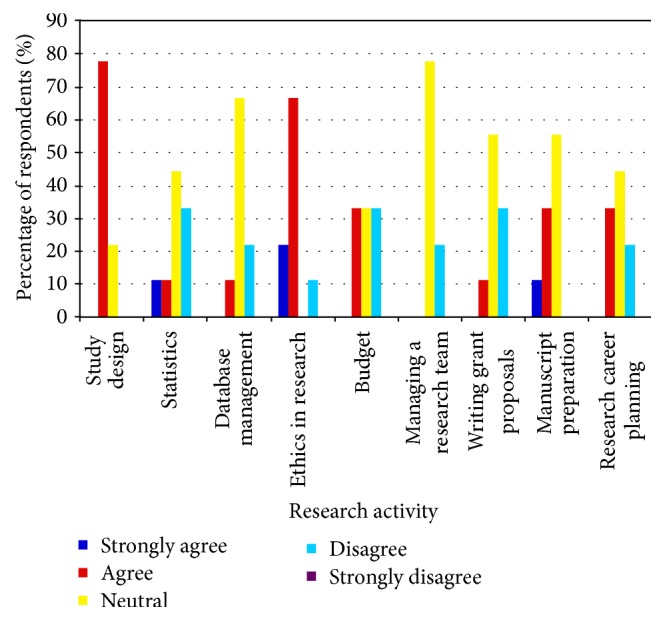
Response by program directors to the survey question: “I believe that graduates of our fellowship are comfortable with performing the following research activities.”

Program directors who responded to the survey represented nine of 21 (43%) adult and pediatric critical care residency programs from across Canada. Fifty-six percent of respondents represented medium-sized programs (six to eight critical care trainees), 22% of respondents represented smaller programs (three to five trainees), and 22% represented larger programs (greater than eight trainees). All program directors noted that more than 50% of their residents were involved in scholarly work and benefited from protected time during residency to pursue research. This protected time ranged from four weeks to over four months throughout the duration of the 2-year training program. The expectation at the end of residency for all but one program for which information was available is that critical care trainees would have completed a study and/or presented a poster/abstract at a meeting. Program directors generally had a positive perception of critical care trainees' readiness to perform various aspects of research by the end of their training but also identified managing a research team and database management as areas of relative weakness (Figure 4).

### 3.5. Research Coordinator Responses

**Figure 5:**
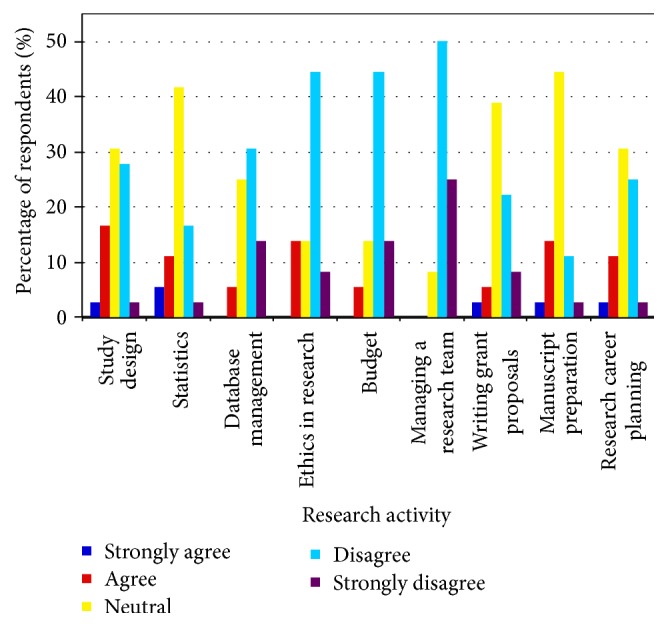
Response of research coordinators to the question: “Within my institution, Critical Care fellows and junior faculty members are well trained in the following areas of research.”

Forty-two percent of surveyed research coordinators stated that they work with critical care trainees and junior faculty. Research coordinators echoed that trainees and junior faculty were least proficient in the areas of managing a research team, developing a budget, and managing a database (Figure 5).

**Figure 6:**
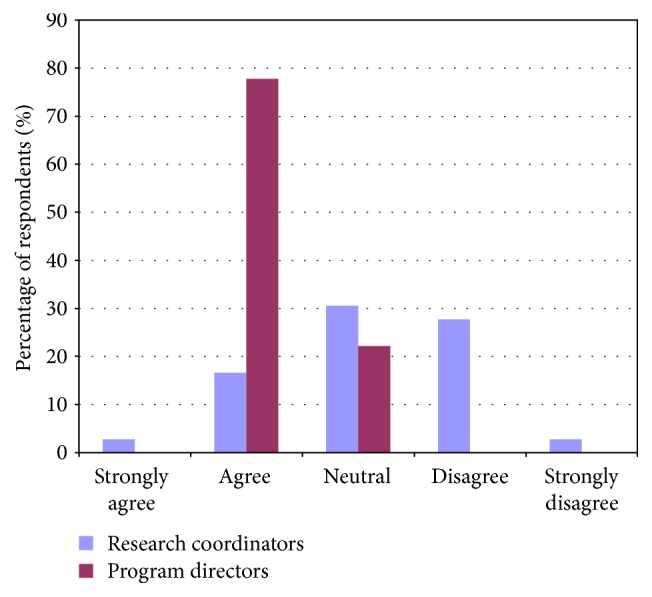
Comparing responses of program directors and research coordinators regarding trainees' abilities in applying appropriate study design when developing a research project.

**Figure 7:**
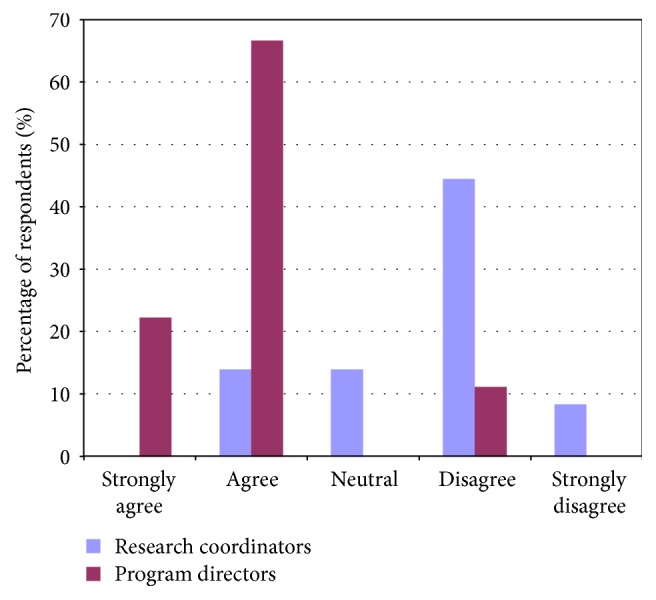
Comparing responses of program directors and research coordinators regarding trainees' abilities in navigating ethics in research.

**Figure 8:**
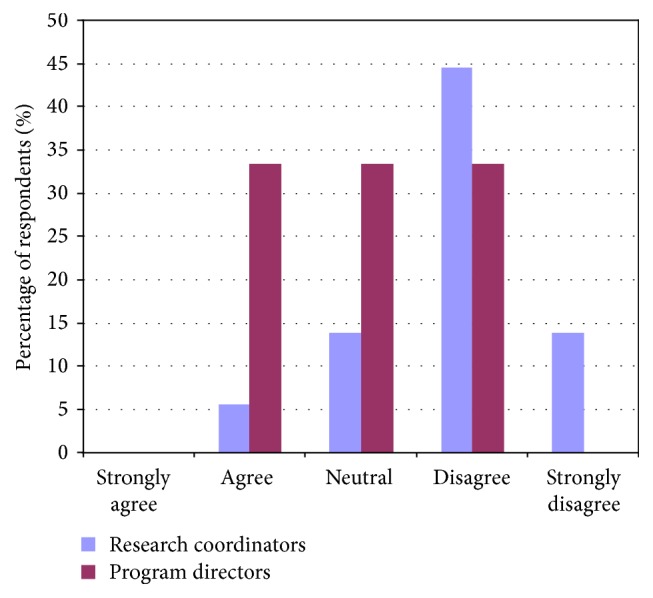
Comparing responses of program directors and research coordinators regarding trainees' abilities in creating and managing a research budget.

Contrary to the feeling of program directors, research coordinators felt that trainees and junior faculty needed to develop proficiency in study design and setting up a research budget. Contrary to the feelings of both program directors and trainees, research coordinators felt that trainees and junior faculty also required more training on ethics in research (Figures 6, 7, and 8).

### 3.6. CCCTG Research Course

Thirty percent of program directors felt that the CCCTG research course was valuable or very valuable, with an additional 20% feeling that it was somewhat valuable. Eighteen percent of faculty surveyed had participated in the CCCTG education program in the past; nonparticipants cited unawareness of the program or inability to get time off clinical work to attend the program as reasons for not attending the course.

Trainees felt that the CCCTG would be a valuable resource in terms of research training, especially for research career mentorship as well as critiquing of research proposals. In terms of information delivery, 40% of program directors and previous attendees felt that longitudinal in-person seminars with a web-based component, such as access to online statistics teaching, would be the most beneficial. However, they recognized the challenge of obtaining time away from clinical duties and monetary perspective.

One theme that came out from many of the qualitative comments was mentorship. Most of the respondents who are involved in research identified having a research supervisor. The level of involvement of the supervisor varied significantly and the amount of support the trainee felt he/she had also varied. Program directors most commonly cited increased availability of mentorship and easier access to statistical support as needed resources for trainees.

## 4. Discussion

Our study demonstrated that trainees and junior faculty members still felt inadequately trained in several research-related areas following their critical care fellowships. This was in contrast with the expressed views of program directors who felt that trainees were generally well prepared for undertaking research. In addition, we found that there were some discrepancies between the areas of perceived need for further training between the trainees/junior faculty and the research coordinators surveyed. The discrepancy between program directors and research coordinators may be due to program directors trying to ensure that trainees learn general skills as a scholar whereas research coordinators are focused specifically upon research. Although almost half of coordinators who responded indicated they worked with trainees and/or junior faculty, the extent to which they worked with them was likely variable. Further work would have to be done to clarify this relationship and its possible link to the opinions of coordinators regarding research educational needs of trainees and junior faculty.

The specific areas of need identified by trainees and junior faculty included database and research team management, how to manage a research team, and how to prepare a research budget, statistical analysis, and grant writing. In addition, research coordinators identified study design and research ethics as further areas in which research training was necessary for critical care trainees and junior faculty. Further study is required to more thoroughly understand why this difference of opinion exists. For example, what are the gaps that the research coordinators perceive trainees have with research ethics? This may prove to be an opportunity for research-focused organizations, such as the CCCTG, which could focus on areas of research training that are not well addressed currently by formal research education. The CCCTG could develop a longitudinal or rotating seminar series based upon these perceived gaps, adding value to the current CCCTG research curriculum. In addition, given their detailed knowledge about the research process, research coordinators may be recruited by the CCCTG as a valuable resource in educating trainees about real-life operational issues related to conducting research.

To date, there are no studies describing critical care research education. Studies related to research education in general have suggested that factors that were most predictive of positive research experiences during training and continuation of a research career included intellectual satisfaction and training grants [7]. With this in mind, the CCCTG could take a role in announcing available grant opportunities and helping trainees with their applications.

Another theme that has become prominent in the literature regarding research education is one of mentorship. Mentors are important not only in the undertaking of a research project, but also in helping trainees shape research careers [7]. Our finding of the importance of mentorship was echoed in the results of a recent survey of critical care medicine trainees. These authors found that trainees expressed a need for more mentorship of nonclinical activities, including research [8]. The CCCTG, along with critical care programs, could consider developing a network of researchers who would be interested in mentoring fellows with various parts of their research and who could also be helpful in research career mentorship. Currently, the CCCTG identifies mentors for junior faculty, but there is a clear need to extend this partnership to trainees and to further formalize the mentorship process. Because a majority of trainees were identified as having a research supervisor, the role of this mentor may not need to be in helping with the finer details of the research project, but more focusing on ways to construct a meaningful research career. It can be assumed that many research skills are honed through experience rather than via formal education, such as managing a research team and creating a budget. This makes longitudinal mentorship even more important as trainees are transitioning from primary work in designing a study to more challenging and managerial aspects of research. Another role for CCCTG mentors would be to expose trainees to researchers who excel in various different styles of research, such as Knowledge Translation, to give trainees a wide example of types of research available.

Even though a majority of trainees were involved in a research project during their critical care residencies, only 60% were interested in pursuing research in their future career. A similar trend was found amongst junior faculty members, with only 20% of those surveyed currently in a research-based position. It is unclear whether this represents the whole group of trainees and junior faculty members, but it would be important to pursue so as to understand better the barriers to a successful research career. It is unclear whether this is due to waning interests, lack of protected time, competing clinical careers, or other barriers. In order to gain a deeper understanding about the data gathered above, hosting focus groups of trainees at their respective sites or via teleconference would be a good adjuvant.

As a majority of respondents felt that a longitudinal research training program would be the most beneficial to improving research knowledge and skills, further work needs to be done to identify how curriculum content could be best disseminated (e.g., online seminars and live webinars) in spite of workload and monetary constraints. Consideration must be given to time available for these opportunities, given all the other responsibilities that critical care trainees have. Currently in Canada, this training is 2 years in length; for people interested in a research career, training beyond these 2 years may be required.

This study's major limitation is the fact that there were differences in representation within the respondent groups. In particular, research coordinators were better represented than critical care trainees. In addition, our absolute number of respondents was small, especially in the trainee and junior faculty categories, yielding a response rate less than the 50–75% desired [9, 10]. Having robust data representing the opinions of junior faculty members would have been valuable as we felt that they would have the most insight into what their needs were transitioning from training to being a clinician and researcher. Future studies are needed to target input from these groups. Further, we do not know to what extent respondents and nonrespondents differ, introducing a potential source of bias into our results. Another limitation would be that faculty in nonacademic centres and community critical care physicians were not included in this study. These physicians may argue that intensive research training during fellowship would not have been necessary or valuable for their career progression. This information would be important to determine whether a dedicated CCCTG research course would be appropriate or necessary for all trainees. Finally, further engagement with program directors is planned regarding how they envision the CCCTG research curriculum complementing their locally available educational opportunities. We plan to create a summary of all the available research educational opportunities from around the country so that individual trainees could potentially access the resources they need to prepare them for a future research career.

## 5. Conclusion

Research education is an important part of training for all critical care physicians. With the information from our survey, we envision a research curriculum that complements training provided locally so that all research education goals are met by the end of training. Although we focused on the needs of critical care trainees in Canada, these data may be more widely applicable to trainees of all postgraduate programs in Canada or internationally.

As it has in the past, the CCCTG can continue to be a valuable resource for the education of current and future critical care trainees to help prepare them for a career in research.

## Supplementary Material

Items contained within the online research education questionnaire sent to critical care residents during this study.

## Figures and Tables

**Table 1:** Number of survey respondents, response rate, and total response rate based on identified position.

Title	Number of surveys sent	Number of respondents	Response rate
Research coordinators	60	38	63%
Program directors	21	9	43%
Junior faculty	40	11	27%
Trainees	114	28	25%

Total	**235**	**86**	**37%**

**Table 2:** Demographics of critical care trainees who responded to the survey.

Description of trainee	Percentage of respondents
Program stream	
(i) Royal College stream	86%
(ii) Clinical Fellow stream^1^	14%

Training program	
(i) Adult	71%
(ii) Pediatric	29%

Specialty prior to critical care	
(i) Anesthesia	18%
(ii) General surgery	7%
(iii) Internal medicine	39%
(iv) Pediatrics	25%
(v) Other (cardiac surgery, emergency)	11%

Prior advanced degree (master's, Ph.D.)	
(i) Yes	14%
(ii) No	86%

^1^Clinical Fellows are generally trainees who come from other countries with the sole purpose of education.

## Appendix

### The Canadian Critical Care Trials Group

Neill Adhikari, M.D. (Sunnybrook Health Sciences Centre), Eyad Althenayan, M.D. (Western University), Patrick Archambault, M.D. (CSSS Alphonse-Desjardins-CHAU Hôtel-Dieu de Lévis), Sean Bagshaw, M.D. (University of Alberta), Andrew Baker, M.D. (St. Michael's Hospital), Ian Ball, M.D. (Western University), Jane Batt, M.D. and Ph.D. (St. Michael's Hospital), Karen Bosma, M.D. (London Health Sciences Centre), Gordon Boyd, M.D. and Ph.D. (Kingston General Hospital), Laurent Brochard, M.D. and H.D.R. (St. Michael's Hospital), Karen Burns, M.D. (St. Michael's Hospital), Jeff Burzynski, M.D. (University of Manitoba Health Sciences Center), François-Martin Carrier (Centre Hospitalier de l'Université de Montréal), Emmanuel Charbonney, M.D. and Ph.D. (CSSSTR (Trois-Rivières) Centre Hospitalier Affilié Universitaire), Michaël Chassé, M.D. and Ph.D. (Centre de Recherche du CHU de Québec), Karen Choong, M.B. (McMaster University), Bryan Coburn, M.D. and Ph.D. (University Health Network-Toronto General Hospital), Deborah Cook, M.D. (McMaster University), Nick Daneman, M.D. (Sunnybrook Health Sciences Centre), Frederick D'Aragon, M.D. and Ph.D. (Centre Hospitalier Universitaire de Sherbrooke), Sonny Dhanani, M.D. (Children's Hospital of Eastern Ontario), Peter Dodek, M.D. (Center for Health Evaluation & Outcome Sciences), John Drover, M.D. (Queen's University), Guillaume Emeriaud, M.D. and Ph.D. (CHU Sainte-Justine), Shane English, M.D. (Ottawa Health Research Institute), and Eddy Fan, M.D. and Ph.D. (Mount Sinai Hospital). Catherine Farrell, M.D. (CHU Sainte-Justine), Niall Ferguson, M.D. (Mount Sinai Hospital), Patricia Fontela, M.D. and Ph.D. (McGill University Health Center), Jennifer Foster, M.D. (London Health Sciences Centre), Rob Fowler, M.D. (Sunnybrook Health Sciences Centre), Alison Fox-Robichaud, M.D. (Hamilton Health Sciences), Charles Francoeur, M.D. (CHU de Québec-Hôpital Enfant-Jésus), Elaine Gilfoyle, M.D. (Alberta Children's Hospital), Martin Girard, M.D. (Centre Hospitalier de l'Université de Montréal), Ronald Gottesman, M.D. (McGill University Health Center), Robert Green, M.D. (Dalhousie University), Donald Griesdale, M.D. (Vancouver General Hospital), Anne-Marie Guerguerian, M.D. and Ph.D. (Hospital for Sick Children), Richard Hall, M.D. (Dalhousie University), Betty Jane Hancock, M.D. (Children's Hospital), Paul Hébert, M.D. (Centre Hospitalier de l'Université de Montréal), Ahmed Hegazy, M.B. (University of Western Ontario), Margaret Herridge, M.D. (University Health Network-Toronto General Hospital), Jamie Hutchison, M.D. (Hospital of Sick Children), Tim Karachi, M.D. (McMaster University), Constantine Karvellas, M.D. (University of Alberta), Brian Kavanagh, M.D. (The Hospital for Sick Children), Michelle Kho, Ph.D. (McMaster University), Niranjan Kissoon, M.D. (British Columbia Children's Hospital), Karen Koo, M.D. (McMaster University), Andreas Kramer, M.D. (Foothills Medical Centre), Arnold Kristoff, M.D. (McGill University Health Center), Jim Kutsogiannis, M.D. (Royal Alexandra Hospital), Jacques Lacroix, M.D. (CHU Sainte-Justine), François Lamontagne, M.D. (Centre Hospitalier de l'Université de Sherbrooke), François Lauzier, M.D. (CHU de Québec, Hôpital de l'Enfant-Jésus), Osama Loubani, M.D. (Dalhousie University), Sheldon Magder, M.D. (McGill University Health Center), John Marshall, M.D. (St. Michael's Hospital), Claudio Martin, M.D. (London Health Sciences Centre), Victoria McCredie, M.D. and Ph.D. (Sunnybrook Health Sciences Centre), Lauralyn McIntyre, M.D. (Ottawa Health Research Institute), James McNally, M.D. (Children's Hospital of Eastern Ontario), Maureen Meade, M.D. (McMaster University), Nav Mehta, M.D. (Health Sciences North), Sangeeta Mehta, M.D. (Mount Sinai Hospital), Tina Mele, M.D. and Ph.D. (London Health Sciences Centre), Kusum Menon, M.D. (Children's Hospital of Eastern Ontario), Srinivas Murthy, M.D. (British Columbia Children's Hospital), John Muscedere, M.D. (Kingston General Hospital), Simon Oczkowski, M.D. (McMaster University), Joe Pagliarello, M.D. (Ottawa Hospital), Melissa Parker, M.D. (McMaster University), Dominique Piquette, M.D. and Ph.D. (Sunnybrook Health Sciences Centre), Bram Rochwerg, M.D. (Juravinski Hospital), Aimee Sarti, M.D. (Ottawa Hospital), Damon Scales, M.D. and Ph.D. (Sunnybrook Health Sciences Centre), Andrew Seely, M.D. (Ottawa Health Research Institute), Jason Shahin, M.D. (McGill University Health Center), Michael Sharpe, M.D. (London Health Sciences Centre), Jeffrey Singh, M.D. (University Health Network-Toronto Western Hospital), Tasnim Sinuff, M.D. and Ph.D. (Sunnybrook Health Sciences Centre), Yoanna Skrobik, M.D. (McGill University Health Center), Maude St-Onge, M.D. and Ph.D. (CHU de Québec, Centre Antipoison du Québec), Asumi Sugiura, M.D. (The Hospital for Sick Children), Eric Sy, M.D. (Regina General Hospital), Jennifer Tsang, M.D. (St. Catharines General Hospital), Marisa Tucci, M.D. (CHU Sainte-Justine), Alexis Turgeon, M.D. (CHU de Québec, Hôpital de l'Enfant-Jésus), Keith Walley, M.D. (St. Paul's Hospital), Matthew Weiss, M.D. (Université Laval), Dave Wensley, M.B. (British Columbia Children's Hospital), David Williamson, Ph.D. (Hôpital du Sacré-Coeur de Montréal), Brent Winston, M.D. (University of Calgary), Davinia Withington, B.M. (McGill University Health Center), Hannah Wunsch, M.D. (Sunnybrook Health Sciences Centre), Ryan Zarychanski, M.D. (Cancer Care Manitoba), and David Zygun, M.D. (University of Alberta).
