# Supplementary material for: The Development of a Critical Care Resident Research Curriculum: A Needs Assessment
Source: Can Respir J. 2016 Aug 16;2016:9795739. doi: 10.1155/2016/9795739 (PMC5004022; doi:10.1155/2016/9795739)
Supplement: Supplementary file 1 — Items contained within the online research education questionnaire sent to critical care residents during this study. [file 9795739.f1.pdf]

Appendix A: Survey Items

Current Fellows

- Background questions
    - How many years of critical care fellowship have you completed? *(1, 2, 3+)*
    - Are you a fellow in a Royal College Program or are you a Clinical Fellow (with external funding) (*Royal College Fellow, Clinical Fellow*)
    - Where is your primary training site? (*List all critical care sites*)
    - What is your primary specialty? (*Medicine, Anaesthesia, General Surgery, Pediatrics, Other: please specify*)
    - Are you training in Adult or Pediatric Critical Care? (*Adult, Pediatric)*
    - Do you have any advanced degrees (Masters or PhD) (*yes/no)*
        - If yes, in what field? (Free text)
    - Are you interested in a research career? (Defined by at least 0.5 FTE dedicated to research) (*yes/no*)
    - Do you already have a job lined up for when you finish? (*yes*/no*)*
        - If yes to above, how much protected non-clinical time (administrative, teaching or research) will you have? (<0.2 FTE, 0.2-0.5 FTE, >0.5 FTE)

- Research Questions
    - Are you currently working on a research project? (*yes/no)*
    - What stage is your project in? (*select one or multiple*)
        - *Protocol development/literature review*
        - *REB application*
        - *Application for external funding*
        - *Patient recruitment/data collection*
        - *Data analysis*
        - *Development of manuscript*
        - *Other* ___________________

    - What aspects of research do you find most challenging?
        - *Protocol development/literature review*
        - *REB application*
        - *Application for external funding*
        - *Patient recruitment/data collection*
        - *Data analysis*
        - *Development of manuscript*
        - *Other* ___________________

    - Do you have an identified research supervisor? (*yes/no)*
        - If yes, was this supervisor assigned or self-selected? (*Assigned/self-selected*)
        - If yes, how involved has your research supervisor been in your research project? (5 point Likert – not at all, somewhat, neutral, involved, very involved)

- What has he/she done to help? (open ended)

- The Canadian Critical Care Trials Group (CCCTG) hosts an annual research education program where members of the CCCTG present on research topics and then provide feedback for fellow research projects.

  - Have you participated in the CCCTG program in the past? *(yes/no)*
  - What would you like to learn about in a research program? Select all that apply
    - *Research methodology/study design*
    - *Statistics*
    - *Database management*
    - *Ethics in research/working with REB*
    - *Developing a budget*
    - *Writing a grant proposal*
    - *Writing a manuscript*
    - *Other – please specify* __________

  - What do you think would be the best way to present material?
    - *In-person*
    - *Web-based*
    - *Combination of in-person and web-based*
  - What length of research training offered by the CCCTG would be ideal?
    - *1 day*
    - *1 week*
    - *Longitudinal over course of fellowship*

- Have you participated in any formal research education in your fellowship other than that offered by the CCCTG? (i.e. organized by your program, outside clinical time, specifically focused on research) *yes/no*
    - If no, was a course offered? (*yes/no*)
      - If a course was offered, why was it declined? (Select all that apply
        - *No interest in research*
        - *Unable to get time off clinical work,*
        - *Have previously taken similar research course*
        - *Already feel proficient in research*
        - *Other – please specify:* ____________

    - If yes, what have you learned about? (select all that apply)
      - *Research methodology/study design*
      - *Statistics*
      - *Database management*
      - *Ethics in research/working with REB*
      - *Developing a budget*
      - *Writing a grant proposal*
      - *Writing a manuscript*

        - *Other – please specify* __________

    - Who was this course taught by
        - Members of the critical-care faculty
        - University-wide program
        - Other – please specify ______________

- Approximately how much total time have you spent on this formal research education?
    - *One afternoon*
    - *One day*
    - *One week*
    - *One month*
    - *Other – please specify* ______________

- Was this protected time? (*yes/no*)

<u>Junior Faculty</u>

- Background:
    - Are you currently in a research position? (at least 0.5 FTE protected) *(yes/no)*
    - Is your research time protected? (*yes/no)*
        - If no, why is it not protected (free text)
    - What type of research are your currently involved in?
        - *Clinical*
        - *Translational/basic science*
        - *Medical education*
        - *Health economics*
        - *Knowledge translation*
        - *Health technology assessment*
        - *Other – please specify* ________________

    - How long ago did you finish clinical ICU training? (1, 2, 3, 4, 5 years)
    - Where was your primary site of critical care training?
        - (Canada: please specify institute, United States: please specify institute, International: please specify country)
    - Do you have any advanced degrees (Masters or PhD) (*yes/no)*
        - If yes, in what field? (Free text)
    - Within your department/division of critical care, what percentage of staff have protected time for research?
- Did you have any formal research education as fellow? (see questions from fellow list)
- Trained enough or not in each area in list (5 point Likert)
- Where/how did you learn about each area?
    - Formal training as fellow
    - During training as part of advanced degree

- Informal training as fellow
    - Learned yourself after started on faculty
    - Other ___________________________
- Roadblocks to starting successful research career? (open ended)
- CCCTG education program:
    - Did you attend? y/n
        - Value of course (now that can look back on what needed to learn about) 5 point Likert
        - List specifics that you learned that have been proven to be useful
        - List specifics that you learned that have been proven not to be useful
    - What role can CCCTG have in fellow research education? (open ended)
    - Expand program:
        - How should it look (like questions on fellow list)
        - What topics should be covered

- What are local research resources that you currently have access to? Select all that apply:
    - Research institute,
    - methods centre,
    - biostatistician,
    - epidemiologist,
    - database management,
    - coordinators (full time in ICU or funded per project)

- Do you currently have a research mentor? (*yes/no)*
        - If yes, was this mentor assigned or self-selected? (*Assigned/self-selected*)
        - How often do you meet with your mentor?
            - Weekly
            - Monthly
            - Biannually
            - Annually
            - Other: please specify ___________

    - How helpful have you found this mentor from a research perspective? (5 point Likert - not at all, somewhat, neutral, helpful, very helpful)

Program Directors

- How many fellows are in your program?
- Do residents participate in any research in your program? y/n
    - If yes, what percentage of the residents?
- Do they have any protected time for research? y/n
    - If yes, how much?
- What are the research expectations in the program? (check all)
    - Present poster/abstract at a meeting
    - Published manuscript
    - Grant funding

    - Completion of study
    - No research expectation
- Is there any formal research training in your program? y/n (see fellow list for questions)
- What resources at your local university are available? (see other list under fellows questions)
- Are there any university or department based funding sources available for fellow-driven research? y/n
- List any resources that you would like to have available for your fellows research education that are not currently available
- Do you currently send your fellows to the CCCTG 1 day course? y/n
    - If yes, do you fund them to go?
- Role of CCCTG in research education of fellows:
    - Any role? (5 point Likert)
    - What topics/resources should CCCTG provide
- Feasibility of sending fellows to: (5 point likert)
    - Current 1 day in person course
    - 1 day web-based course
    - Week long in-person course
    - Week long web-based course
    - Longitudinal in-person course
    - Longitudinal web-based course
    - Longitudinal combination in-person and web-based course

Trials group members

- background:
    - In a research position? (at least 0.5 FTE protected)
    - Research Interests (open-ended) – clinical, translational, education, health economics, knowledge translation and health technology assessment, other?
    - Is this time actually being protected?
        - If not, why? (open ended)
    - How long ago did you finish clinical ICU training
    - Where?
    - Do you have an advanced degree (Masters, PhD)? In what?
    - Within department/division of critical care, % other staff who have protected time with a primary research focus
- Do you teach in a formal research course at your local site?
- Current local research resources (check all apply):
    - Research institute, methods centre, biostatistician, epidemiologist, database management, coordinators (full time in ICU or funded per project)
- Are you involved in mentorship of residents or junior faculty?
- What areas of research do you find mentees to have most difficulty?
        - Research methodology/study design
        - Statistics
        - Database management
        - Ethics in research/working with REB
        - Developing a budget
        - Writing a grant proposal

        - Writing a manuscript
        - Other _______________________
- Have you presented at or attended the CCCTG research day?
- If yes, how useful did you find it (Likert scale)
- What additional topics/resources should the CCCTG training provide?
- Feasibility of: (5 point likert)
    - Current 1 day in person course
    - 1 day web-based course
    - Week long in-person course
    - Week long web-based course
    - Longitudinal in-person course
    - Longitudinal web-based course
    - Longitudinal combination in-person and web-based course

<u>Research Coordinators</u>

- Do you currently work with fellows on their research project? y/n
- Do you currently work with junior critical care faculty on their research projects? y/n (less than 5 years in practice)
- What are trainees trained well on in your institution with respect to research? (list from fellows questions)
- What do they need more training on? (list from fellows questions)
